# Exploring the impact of PFAS exposure and sleep duration on kidney stone formation in the U.S. population

**DOI:** 10.3389/fpubh.2025.1606191

**Published:** 2025-06-04

**Authors:** Jianbai Chen, Zhiming Zhang, Gongquan Xu, Qisheng Tang, Zhiyong Nie, Jianxin Qiu, Xiaoping Gao

**Affiliations:** Department of Urology, Tangdu Hospital, Fourth Military Medical University, Xi'An, Shaanxi, China

**Keywords:** PFAS, perfluoroalkyl substances, kidney stones, sleep duration, environmental exposure, metabolic disruption

## Abstract

**Background:**

Perfluoroalkyl substances (PFAS), including perfluorooctane sulfonate (PFOS), are persistent environmental pollutants with potential nephrotoxic effects. Concurrently, sleep duration has been implicated in metabolic dysregulation, influencing kidney function. While individual studies have examined the effects of PFAS exposure and sleep duration on kidney health, their combined impact on kidney stone formation remains largely unexplored.

**Methods:**

We conducted a cross-sectional study using data from the National Health and Nutrition Examination Survey (NHANES) 2013-2016. PFAS exposure was assessed through serum concentrations of multiple perfluoroalkyl compounds. Sleep duration was self-reported and categorized as <7 h or ≥7 h. Kidney stone status was determined through self-reported medical diagnoses. Multivariable logistic regression models were used to examine associations between PFAS exposure, sleep duration, and kidney stone formation, adjusting for demographic and lifestyle confounders. Non-restrictive cubic spline (RCS) analysis was employed to assess potential non-linear relationships.

**Results:**

Among 1,263 participants, 551 (43.6%) reported a history of kidney stones. Higher serum concentrations of PFDE, PFHxS n-PFOS, and Sm-PFOS were significantly associated with increased odds of kidney stone formation (*p* < 0.05). Participants with sleep duration <7 h had a 1.03-fold higher risk of kidney stones (95% CI: 1.01–1.10, *p*= 0.007). RCS analysis identified non-linear dose-response relationships for several PFAS compounds suggesting threshold effects. Interaction analysis revealed a synergistic effect between PFAS exposure and insufficient sleep, amplifying kidney stone risk.

**Conclusion:**

Our findings suggest that both PFAS exposure and insufficient sleep independently contribute to kidney stone formation, with evidence of a combined exacerbating effect. These results underscore the importance of addressing environmental exposures and lifestyle factors in kidney stone prevention strategies.

## 1 Introduction

Perfluoroalkyl substances (PFAS) and perfluorooctane sulfonate (PFOS) are a group of synthetic chemicals widely used in various industrial and consumer products due to their unique water- and oil-repellent properties (1). These substances are highly resistant to degradation, leading to their persistence in the environment and their accumulation in both wildlife and human populations (2). Over time, PFAS/PFOS have been found to enter the human body primarily through contaminated drinking water, food, air, and consumer products (3). Their stability and bioaccumulation make them a significant concern in environmental health studies, particularly given their potential for long-term adverse health effects (4). Research on PFAS/PFOS exposure has linked these substances to a variety of health problems, such as liver damage, immune system disruption, and endocrine disorders (5, 6). However, the precise mechanisms through which PFAS/PFOS impact kidney function, especially their role in the formation of kidney stones, are still under investigation.

Kidney stones, or renal calculi, are a prevalent and painful condition that results from the crystallization of salts and minerals within the kidneys (7). The formation of kidney stones is influenced by several factors, including genetic predisposition, dietary habits, fluid intake, and metabolic disorders (8, 9). In addition to these well-known risk factors, recent studies suggest that sleep duration plays a significant role in kidney stone formation, with both insufficient and excessive sleep being linked to metabolic disruptions that affect mineral balance (10). Both insufficient sleep and excessive sleep have been associated with disruptions in metabolic pathways that regulate mineral balance and kidney function, particularly with regard to the metabolism of calcium, phosphate, and oxalate (11, 12). Specifically, insufficient sleep has been shown to lead to excessive calcium excretion, while prolonged sleep may disrupt the regulation of phosphate and oxalate metabolism in the kidneys (13). These disruptions may contribute to an increased risk of kidney stone formation. Despite growing evidence linking sleep patterns to kidney health, the precise mechanisms through which sleep duration affects kidney stone formation remain unclear and warrant further exploration (14).

While the individual effects of PFAS/PFOS exposure and sleep duration on kidney health have been studied in isolation, the combined impact of these two factors on kidney stone risk remains largely unexplored. It is plausible that long-term exposure to PFAS/PFOS could compromise renal function and enhance the susceptibility to kidney stone formation, especially when coupled with disrupted sleep patterns (15). Furthermore, the interaction between environmental pollutants like PFAS/PFOS and lifestyle factors such as sleep duration could result in synergistic effects that exacerbate kidney stone risk. Understanding how these two factors work together is crucial for developing comprehensive strategies to mitigate kidney stone formation, particularly in populations at higher risk due to prolonged exposure to environmental pollutants or poor sleep hygiene.

This study aims to fill this gap by investigating the combined effects of PFAS/PFOS exposure and sleep duration on kidney stone formation, which has not been extensively studied. The research will utilize data from the National Health and Nutrition Examination Survey (NHANES), a comprehensive dataset that includes information on PFAS/PFOS concentrations, sleep duration, and the prevalence of kidney stones in the general population (16). By analyzing these factors alongside potential confounders such as age, sex, diet, and physical activity, this study will provide a clearer understanding of how environmental exposures and lifestyle factors contribute to kidney stone formation. The findings could help identify vulnerable populations, inform preventive strategies, and support public health policies to reduce kidney stone prevalence, especially in areas with high PFAS/PFOS exposure.

Given the growing prevalence of kidney stones worldwide and the widespread exposure to PFAS/PFOS, it is important to investigate how these environmental and lifestyle factors interact to impact kidney health. The findings from this study may offer valuable insights for preventive strategies and help shape future policies focused on reducing both environmental pollutants and improving sleep hygiene.

## 2 Methods

### 2.1 Study population

**Figure 1:**
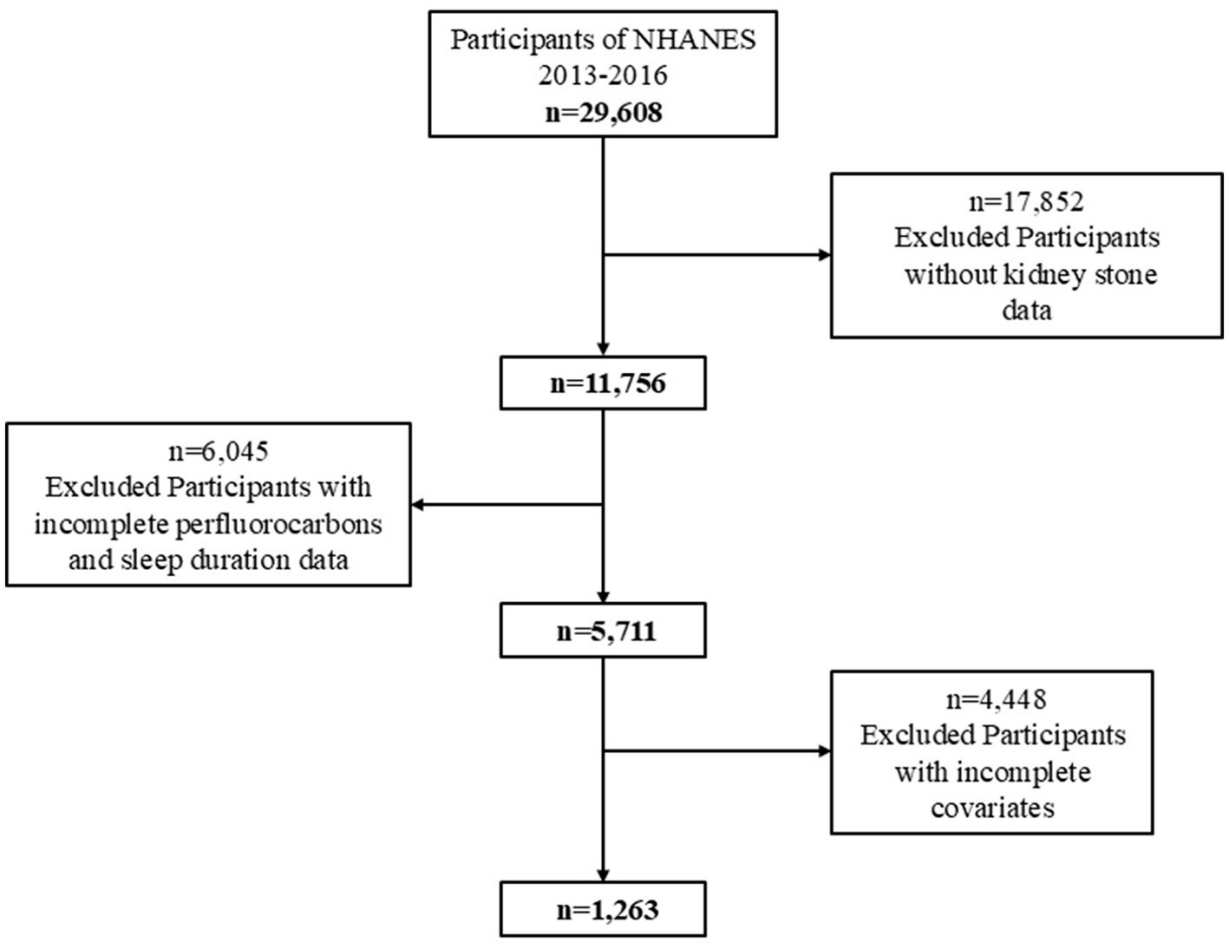
Flowchart of the screening process for the study population.

This study utilized data from the National Health and Nutrition Examination Survey (NHANES) 2013–2016, a cross-sectional survey designed to assess the health and nutritional status of the U.S. population. Initially, 29,608 participants were included in the NHANES dataset. Participants were excluded based on the following criteria: (1) individuals missing data on kidney stone status (*n* = 17,852), (2) individuals with missing data on perfluoroalkyl substances (PFAS) exposure and sleep duration (*n* = 6,045), and (3) individuals missing covariate information (*n* = 6). This led to a final analytical sample of 1,263 participants (Figure 1).

### 2.2 Exposure and outcome variables

#### 2.2.1 PFAS exposure

PFAS exposure was measured using blood concentrations of various perfluoroalkyl compounds, including perfluorodecanoic acid (PFDE), perfluorohexane sulfonate (PFHxS), perfluorononanoic acid (PFNA), perfluorooctanoic acid (n-PFOA), perfluorooctane sulfonate (n-PFOS), and sulfonated PFOS (Sm-PFOS). PFAS exposure was measured using blood concentrations of various perfluoroalkyl compounds, which reflect short-term exposure (weeks to months). The concentrations of these compounds were measured in nanograms per milliliter (ng/ml), and total PFAS exposure was calculated as the sum of all individual PFAS compounds (17).

#### 2.2.2 Sleep duration

Sleep duration was self-reported by participants and categorized into two groups: <7 and 7 h or more. This classification allowed for the assessment of both insufficient sleep (<7 h) and sufficient sleep (7 h or more) in relation to kidney stone formation.

#### 2.2.3 Kidney stone status

Kidney stone status was determined through self-reporting of a medical diagnosis of kidney stones, corroborated by imaging studies when available (18).

### 2.3 Covariates

Covariates included potential confounders such as gender, age, race, education level, poverty income ratio (PIR), alcohol use, hypertension, body mass index (BMI), high-density lipoprotein (HDL) cholesterol, and total cholesterol. These variables were selected based on prior research indicating their potential influence on kidney stone risk.

### 2.4 Statistical analysis

#### 2.4.1 Descriptive statistics

Baseline characteristics of the study population were summarized using means and standard deviations for continuous variables and frequencies and percentages for categorical variables. Differences in baseline characteristics between individuals with and without kidney stones were tested using independent *t*-tests for continuous variables and chi-square tests for categorical variables. Regarding the use of survey weights, we followed NHANES official guidelines by incorporating the appropriate sampling weights to adjust for the complex survey design and ensure the representativeness of the U.S. population. Specifically, we used the Mobile Examination Center (MEC) examination weights (wtmec4yr) as recommended by NHANES protocols for our analysis.

#### 2.4.2 Logistic regression

To examine the relationship between PFAS exposure, sleep duration, and kidney stone formation, weighted logistic regression analyses were conducted using SPSS 27.0. Three models were used to account for different levels of adjustment:

- *Model 1*: unadjusted, including only the exposure variables (PFAS and sleep duration) and the outcome (kidney stone status).
- *Model 2*: adjusted for gender and age.
- *Model 3*: fully adjusted for gender, age, race, education level, PIR, alcohol use, hypertension, BMI, HDL cholesterol, and total cholesterol.

Odds ratios (ORs) and 95% confidence intervals (CIs) were estimated for each exposure variable in relation to kidney stone formation.

#### 2.4.3 Non-restrictive cubic spline (RCS) analysis

To examine the dose-response relationship between PFAS exposure and kidney stone formation, non-restrictive cubic spline (RCS) analysis was employed using R 4.4.3. This technique allows for the modeling of non-linear relationships between PFAS concentrations and kidney stone risk, providing a more flexible representation of the data compared to traditional linear models. The RCS method enables the detection of threshold effects and the assessment of potential dose-response relationships, making it particularly useful for exploring complex environmental exposure effects on health outcomes. The cubic spline was fitted with three knots, chosen based on model diagnostics and previous research, to ensure an appropriate balance between model fit and complexity. We also performed goodness-of-fit tests using Akaike Information Criterion (AIC) values to validate the model selection.

#### 2.4.4 Subgroup and interaction analysis

Subgroup analyses were conducted to evaluate potential effect modification by demographic factors, such as age, gender, and race. These analyses aimed to determine whether the associations between PFAS exposure, sleep duration, and kidney stone risk varied across different population subgroups.

Additionally, interaction terms between PFAS exposure and sleep duration were included in the logistic regression models to assess potential synergistic effects on kidney stone formation. This interaction analysis helped identify whether the combined influence of PFAS exposure and sleep duration on kidney stone risk was stronger than the individual effects of each factor.

### 2.5 Ethical considerations

The study was conducted in accordance with the ethical standards set forth by the NHANES protocols, with all participants providing informed consent prior to their inclusion in the survey. Data collection for the NHANES was approved by the National Center for Health Statistics (NCHS) Research Ethics Review Board (ERB) under Continuation of Protocol #2011-17. All participants provided written informed consent prior to data collection.

## 3 Results

### 3.1 Baseline characteristics

The baseline characteristics of the study population, stratified by kidney stone status, are presented in Table 1. Of the 1,263 participants included, 551 (43.6%) reported a history of kidney stones. Participants with kidney stones were significantly older (mean age 51.65 ± 17.20 years) compared to those without kidney stones (mean age 47.31 ± 17.35 years, *p* < 0.001). Males had a higher prevalence of kidney stones than females [259 (20.5%) vs. 292 (23.1%), *p* < 0.001].

**Table 1:** Baseline characteristics of the study participants.

**Characteristics**	**Overall**	**Kidney stone**		***p*-value**
		**Yes**	**No**	
*n*	1,262	551	711	
Age, years	49.21 ± 17.41	51.65 ± 17.20	47.31 ± 17.35	<0.001
Gender, *n* (%)				<0.001
Female	604 (47.9%)	292 (23.1%)	312 (24.7%)	
Male	658 (52.1%)	259 (20.5%)	399 (31.6%)	
Race, *n* (%)				<0.001
Mexican American	217 (17.2%)	102 (8.1%)	115 (9.1%)	
Other Hispanic	155 (12.3%)	62 (4.9%)	93 (7.4%)	
Non-Hispanic Black	432 (34.2%)	160 (12.7%)	272 (21.6%)	
Non-Hispanic White	293 (23.2%)	162 (12.8%)	131 (10.4%)	
Other races	165 (13.1%)	100 (7.9%)	65 (5.2%)	
Education, *n* (%)				<0.001
Less than 9th grade	126 (10.0%)	57 (4.5%)	69 (5.5%)	
9–11th grade	141 (11.2%)	73 (5.8%)	68 (5.4%)	
High school graduate	282 (22.3%)	133 (10.5%)	149 (11.8%)	
Some college or AA degree	406 (32.2%)	174 (13.8%)	232 (18.4%)	
College graduate or above	307 (24.3%)	114 (9.0%)	193 (15.3%)	
PIR, *n* (%)				<0.001
≤1	285 (22.6%)	165 (13.1%)	120 (9.5%)	
1–3	539 (42.7%)	220 (17.4%)	539 (42.7%)	
>3	438 (34.7%)	166 (13.2%)	272 (21.6%)	
Alcohol use, *n* (%)				<0.001
Yes	888 (70.4%)	376 (29.8%)	512 (40.6%)	
No	374 (29.6%)	175 (13.9%)	199 (15.8%)	
Hypertension, *n* (%)				<0.001
Yes	310 (24.6%)	161 (12.8%)	149 (11.8%)	
No	952 (75.4%)	390 (30.9%)	562 (44.5%)	
BMI				<0.001
≤19.9	41 (3.2%)	10 (0.8%)	31 (2.5%)	
19.9–25	303 (24.0%)	212 (16.8%)	91 (7.2%)	
25–30	403 (31.9%)	155 (12.3%)	248 (19.7%)	
≥30	515 (40.8%)	295 (23.4%)	220 (17.4%)	
HDL-cholesterol (mg/dl)	54.37 ± 17.31	51.37 ± 16.75	56.70 ± 17.40	
Total cholesterol (mg/dl)	190.23 ± 41.09	187.74 ± 40.40	192.16 ± 41.55	

Racial distribution showed notable differences, with non-Hispanic Black participants accounting for a higher proportion in the kidney stone group (29.0%) compared to those without kidney stones (21.6%, *p* < 0.001). Participants with kidney stones were more likely to have lower education levels (less than a college degree), lower poverty income ratio (PIR ≤ 1), and a higher prevalence of alcohol consumption (*p* < 0.001 for all).

Additionally, hypertension was more common in individuals with kidney stones (29.2 vs. 15.7%, *p* < 0.001), and obesity (BMI ≥ 30) was significantly associated with kidney stone presence (53.5 vs. 30.9%, *p* < 0.001). Furthermore, participants with kidney stones had lower HDL cholesterol levels (51.37 ± 16.75 vs. 56.70 ± 17.40 mg/dl) and slightly lower total cholesterol (187.74 ± 40.40 vs. 192.16 ± 41.55 mg/dl; Table 1). In terms of PFAS exposure, participants with kidney stones demonstrated significantly higher serum concentrations of PFDE, PFHxS, PFNA, n-PFOS, and Sm-PFOS compared to those without kidney stones (*p* < 0.05 for all), as shown in Table 2.

**Table 2:** Description of perfluoroalkyl levels among participants.

**Characteristics**	**Overall**	**Kidney stone**		***p*-value**
		**No**	**Yes**	
*N* (average ± SD)	1,262	711	551	
**Individual PFAS (ng/ml)**				
PFDE	0.26 ± 0.48	0.23 ± 0.27	0.30 ± 0.65	<0.001
PFHxS	1.75 ± 1.93	1.60 ± 1.39	1.96 ± 2.44	<0.001
PFNA	0.81 ± 0.73	0.79 ± 0.65	0.83 ± 0.83	0.023
n-PFOA	1.92 ± 1.71	1.95 ± 1.63	1.87 ± 1.80	0.298
n-PFOS	5.51 ± 7.01	4.69 ± 3.93	6.58 ± 9.51	<0.001
Sm-PFOS	2.17 ± 1.91	1.99 ± 1.57	2.40 ± 2.27	<0.001
Total PFAS	12.42 ± 10.70	11.25 ± 7.14	13.94 ± 13.88	<0.001

### 3.2 Association of PFAS exposure and sleep duration with kidney stone formation

Weighted logistic regression analyses (Table 3) revealed that higher levels of PFDE (OR = 1.50, 95% CI: 1.13–1.98, *p* = 0.005), PFHxS (OR = 1.09, 95% CI: 1.02–1.16, *p* = 0.009), n-PFOS (OR = 1.04, 95% CI: 1.02–1.06, *p* < 0.001), and Sm-PFOS (OR = 1.08, 95% CI: 1.00–1.16, *p* = 0.049) were positively associated with the odds of kidney stone occurrence in fully adjusted models (Model 3). In addition, total PFAS exposure remained significantly associated with kidney stone risk (OR = 1.02, 95% CI: 1.01–1.04, *p* < 0.001).

**Table 3:** Weighted logistic regression analyses of association between the C-reactive protein to lymphocyte ratio and depression.

**Exposure**	**Model 1**		**Model 2**		**Model 3**	
	**OR 95% CI**	***p*** **-value**	**OR 95% CI**	***p*** **-value**	**OR 95% CI**	***p*** **-value**
PFDE	1.38 (1.06, 1.79)	0.018	1.29 (1.00, 1.67)	0.050	1.50 (1.13, 1.98)	0.005
PFHxS	1.11 (1.04, 1.18)	0.002	1.06 (0.99, 1.12)	0.080	1.09 (1.02, 1.16)	0.009
PFNA	1.09 (0.93, 1.27)	0.277	0.99 (0.84, 1.16)	0.876	1.08 (0.91, 1.27)	0.388
n-PFOA	0.97 (0.91, 1.04)	0.429	0.92 (0.86, 0.99)	0.036	0.99 (0.92, 1.07)	0.740
n-PFOS	1.05 (1.03, 1.07)	<0.001	1.03 (1.01, 1.05)	0.003	1.04 (1.02, 1.06)	<0.001
Sm-PFOS	1.12 (1.05, 1.19)	<0.001	1.04 (0.97, 1.11)	0.291	1.08 (1.00, 1.16)	0.049
Total PFAS	1.03 (1.01, 1.04)	<0.001	1.01 (1.00, 1.03)	0.022	1.02 (1.01, 1.04)	<0.001
**Sleep duration**						
<7 h	1.03 (1.01, 1.11)	0.047	1.03 (1.00, 1.05)	0.050	1.03 (1.01, 1.10)	0.007
≥7 h	Reference		Reference		Reference	
*p* for trend		<0.001	<0.001		<0.001	

Model 1: no covariates were adjusted.

Model 2: age, sex, and race were adjusted.

Model 3: age, sex, race, education level, marital status, BMI, PIR, smoking status, alcohol status, diabetes status, hypertension status, hyperlipidemia status was adjusted.

95% CI, 95% confidence interval.

Sleep duration <7 h was also significantly associated with an increased risk of kidney stone formation across all models. In the fully adjusted model (Model 3), participants with shorter sleep duration had a 1.03-fold higher risk (95% CI: 1.01–1.10, *p* = 0.007) compared to those with sleep duration of ≥7 h. To assess the public health relevance of insufficient sleep in relation to kidney stone formation, the population attributable fraction (PAF) was calculated. The PAF for insufficient sleep in relation to kidney stone formation is estimated to be 0.49%.

### 3.3 Non-linear dose–response relationship (RCS analysis)

**Figure 2:**
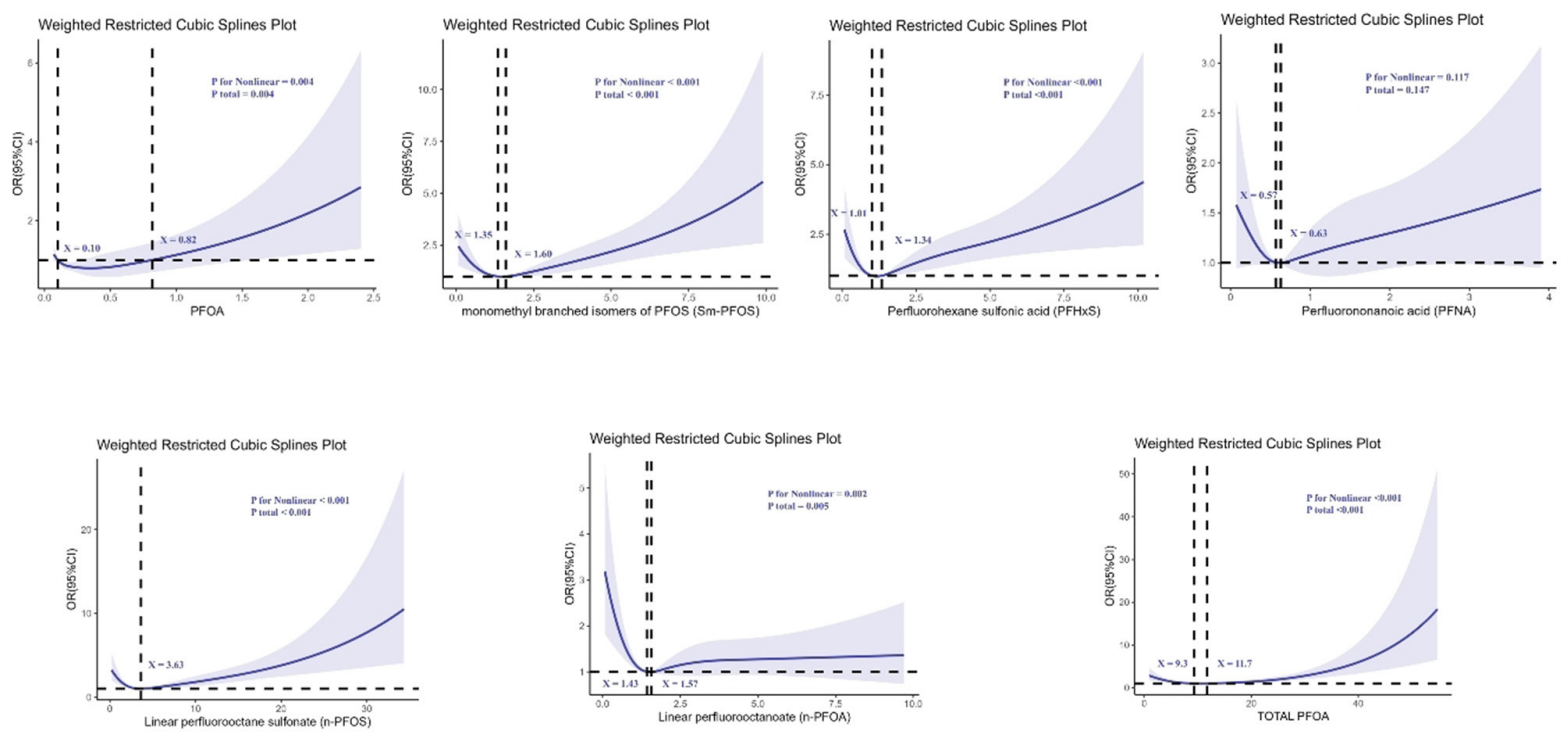
Determination of the association between perfluoroalkyl and polyfluoroalkyl substances and kidney stone by restricted cubic spline (RCS) regression analysis.

Non-restrictive cubic spline (RCS) analyses further revealed significant non-linear associations between certain PFAS compounds and kidney stone risk (all *p* for non-linearity <0.05), as illustrated in Figure 2.

For n-PFOA, the curve demonstrated a clear non-linear relationship, with two turning points at serum concentrations of 1.43 and 1.57 ng/ml, indicating that risk increased sharply beyond these levels. For n-PFOS, a non-linear relationship was observed with an inflection point at 3.63 ng/ml, beyond which the risk of kidney stones increased rapidly. PFHxS also showed a non-linear pattern, with turning points at 1.01 and 1.34 ng/ml. The risk rose notably between these values. In addition, PFOA exhibited two turning points at 0.10 and 0.82 ng/ml, suggesting that even low-level exposure could be associated with increasing risk. For Sm-PFOS, inflection points were observed at 1.35 and 1.60 ng/ml, with the kidney stone risk increasing significantly beyond these thresholds. Total PFOA exposure also presented a non-linear association, with thresholds at 9.3 and 11.7 ng/ml, indicating a steep increase in kidney stone risk beyond these concentrations. Overall, these findings indicate that certain PFAS compounds exhibit threshold effects, beyond which the risk of kidney stones escalates disproportionately.

**Figure 3:**
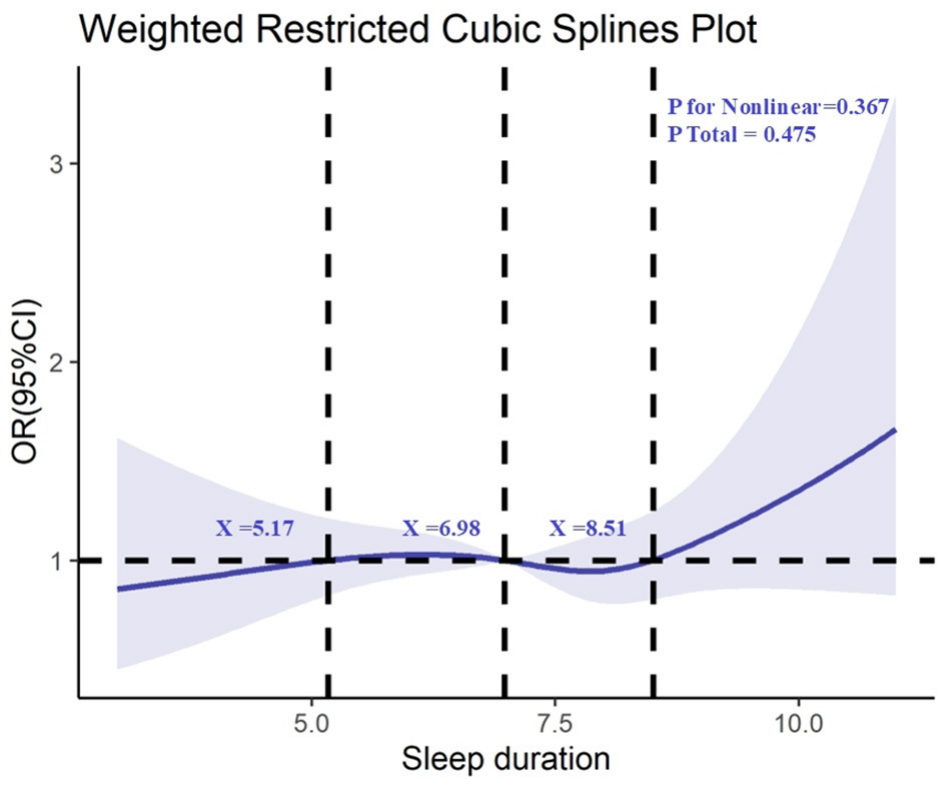
Determination of the association between sleep duration and kidney stone by restricted cubic spline (RCS) regression analysis.

Furthermore, we conducted RCS analysis to explore the relationship between sleep duration and kidney stones, which revealed no significant non-linear association between sleep duration and the risk of kidney stones (non-linearity test *p* = 0.367), as shown in Figure 3.

**Figure 4:**
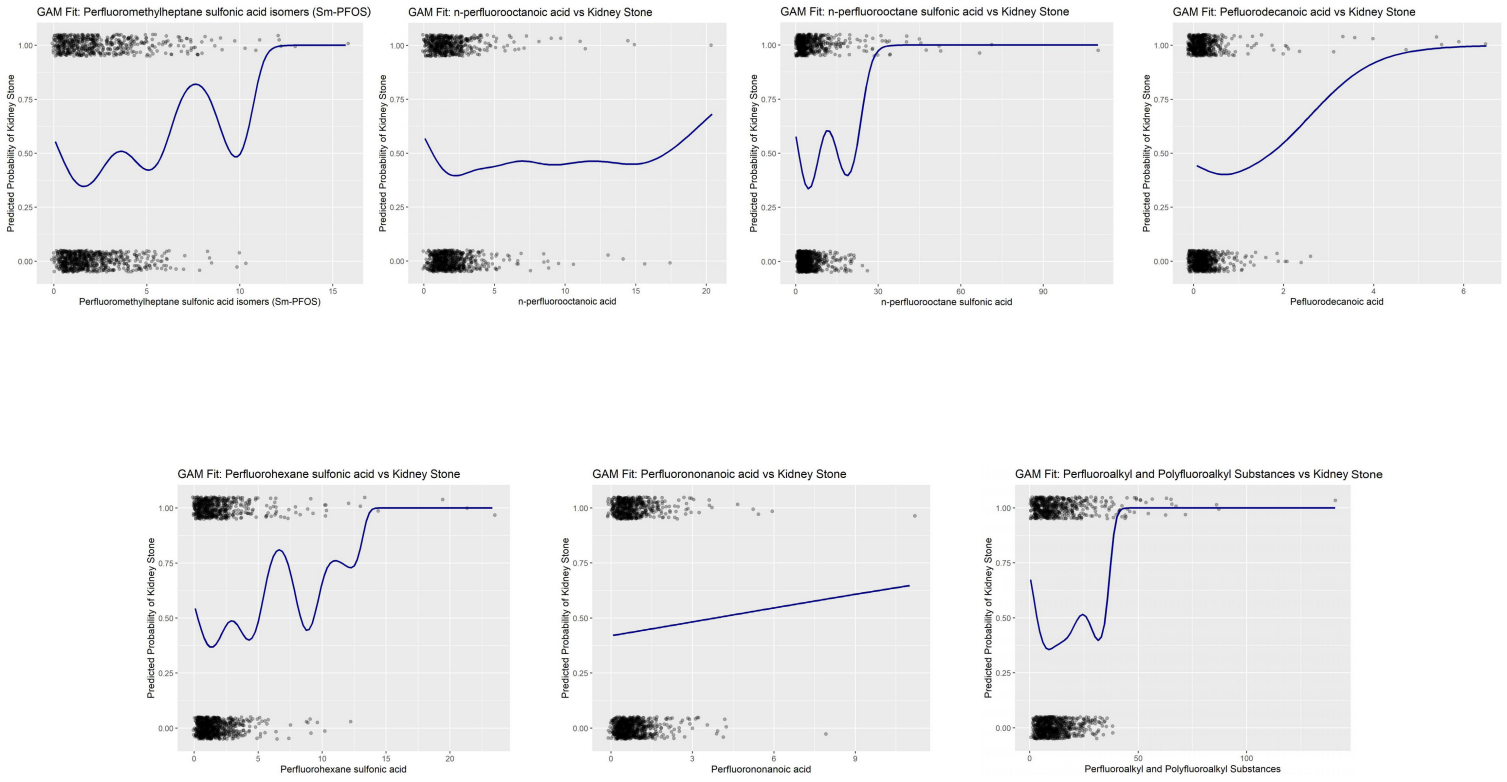
Goodness-of-fit validation of cubic spline generalized additive models (GAMs) for PFAS exposure and kidney stone risk.

To validate the model selection and ensure the robustness of the findings, we performed goodness-of-fit tests using AIC values, which are shown in Figure 4. These tests confirm the appropriateness of the cubic spline model for capturing the dose-response relationships between PFAS exposure and kidney stone formation.

### 3.4 Subgroup and interaction analyses

Subgroup analyses demonstrated that the association between PFAS exposure and kidney stone formation was more pronounced in males, older participants, and non-Hispanic Black individuals.

**Figure 5:**
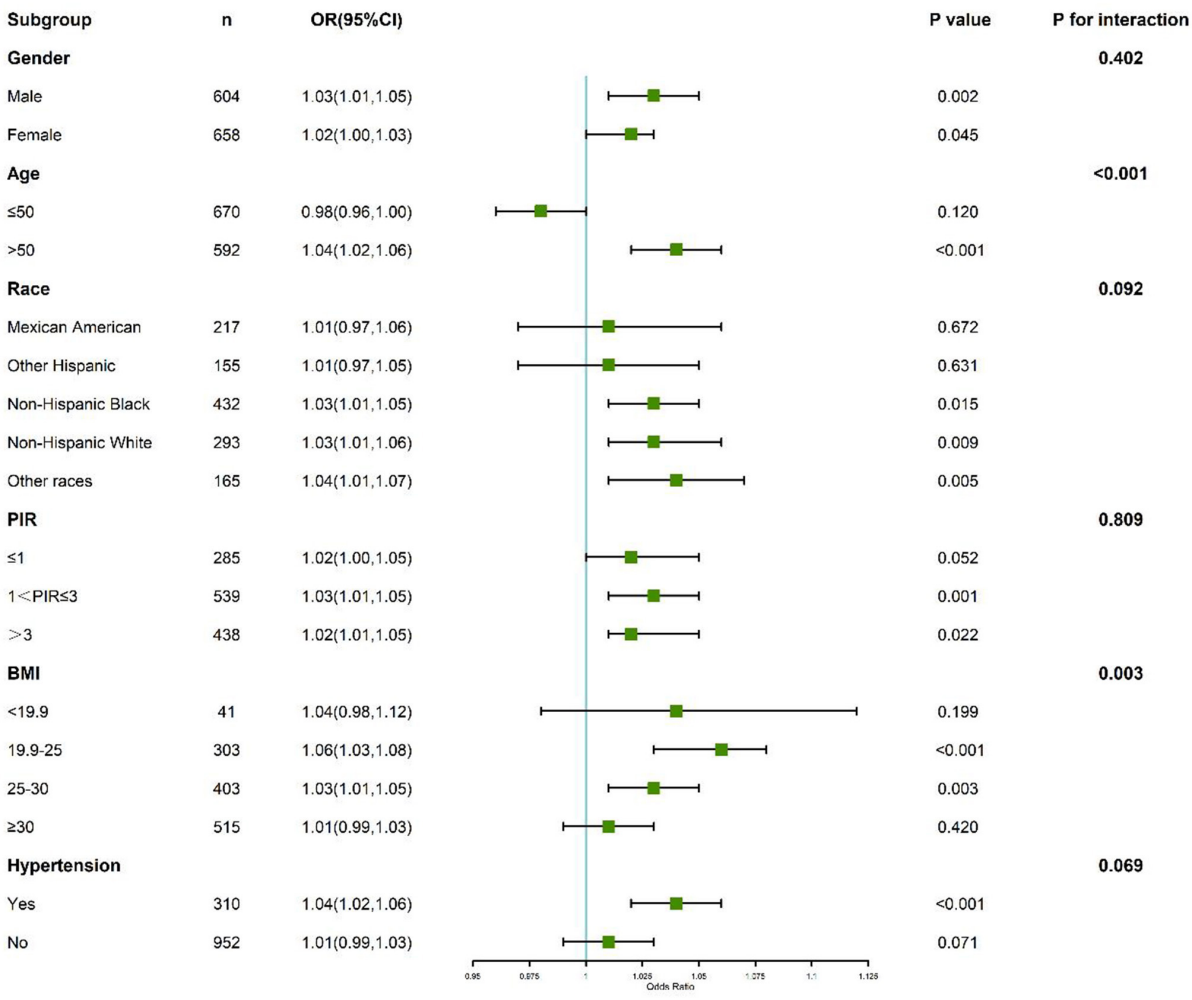
Subgroup and interaction analyses of the association between perfluoroalkyl and polyfluoroalkyl substances, sleep duration, and kidney stone.

Further interaction analyses revealed a significant interaction between PFAS exposure and sleep duration. Participants with both higher PFAS levels and sleep duration <7 h exhibited an amplified risk of kidney stone formation compared to those with only one risk factor. The synergistic effect was particularly evident for PFHxS and Sm-PFOS (*p* for interaction <0.05). These findings suggest that insufficient sleep may exacerbate the nephrotoxic impact of PFAS exposure on kidney stone formation (Figure 5).

## 4 Discussion

This study provides compelling evidence that both perfluoroalkyl substances (PFAS) exposure and insufficient sleep are independently associated with an increased risk of kidney stone formation in the U.S. population. Additionally, our findings suggest that there is a synergistic effect between PFAS exposure and sleep duration, with the combination of high PFAS levels and inadequate sleep (<7 h) leading to a particularly elevated risk of kidney stones. Notably, the results revealed non-linear dose-response relationships between specific PFAS compounds and kidney stone risk, further complicating the effects of environmental pollutants on human health. These findings shed light on the complex interplay between environmental exposures and lifestyle factors in contributing to the rising prevalence of kidney stones (19).

Our results align with existing literature that links PFAS exposure to various health risks, including kidney function impairment and metabolic disruption. PFAS are well-known for their ability to persist in the human body due to their bioaccumulative properties, and their effects on renal function have been previously explored in studies examining liver damage, endocrine disruption, and immune system dysfunction (20, 21). However, this study is novel in its integration of PFAS exposure with sleep duration as a dual environmental and lifestyle risk factor for kidney stone formation. While individual effects of PFAS and sleep duration have been studied in isolation, the combined impact of these two factors has not been extensively explored, making this study a valuable contribution to understanding how multiple risk factors can act synergistically.

The non-linear relationships observed in the non-restrictive cubic spline (RCS) analysis provide additional insight into the mechanisms underlying PFAS exposure and kidney stone formation. The identified threshold effects suggest that even low levels of PFAS, such as n-PFOA and Sm-PFOS, can significantly increase the risk of kidney stones, challenging the assumption that only high-level exposures are harmful. These findings are particularly relevant for public health policies aimed at regulating PFAS contamination in drinking water, food, and consumer products (22). The discovery of these threshold effects could prompt the establishment of more stringent guidelines for PFAS exposure, especially in areas with high contamination levels.

The role of sleep duration in kidney stone formation offers another important avenue for public health intervention. Our findings suggest that insufficient sleep (<7 h) significantly contributes to kidney stone risk, likely due to its influence on calcium metabolism, urinary calcium excretion, and the regulation of other minerals such as phosphate and oxalate (23, 24). The PAF of 0.49% underscores the potential public health impact of addressing insufficient sleep, as a meaningful proportion of kidney stone cases may be preventable through population-level interventions. These findings emphasize optimizing sleep duration as a modifiable risk factor and highlight the importance of promoting sleep hygiene in preventive strategies. Sleep disorders are known to disrupt various metabolic pathways, and our results further emphasize the need for better sleep hygiene as a preventive measure for kidney stone formation (25, 26). Sleep deprivation has been linked to an increase in the production of stress hormones, which could potentially exacerbate mineral imbalances in the body, leading to the crystallization of minerals in the kidneys. Conversely, excessive sleep (>8 h) may also contribute to kidney stone formation through similar metabolic disruptions, suggesting that an optimal sleep duration of 7–8 h is critical for maintaining kidney health.

The mechanisms linking PFAS exposure to kidney stones are likely multifactorial. PFAS compounds are known to disrupt hormonal regulation, including the secretion of thyroid and sex hormones, which are involved in calcium and phosphate metabolism (27, 28). Studies have shown that PFAS exposure can lead to altered calcium homeostasis, increasing urinary calcium levels, which is a key factor in kidney stone formation (29). Furthermore, PFAS may also influence the regulation of other minerals such as oxalate, which plays a crucial role in the crystallization process of kidney stones (30). Additionally, PFAS-induced inflammation and oxidative stress may further damage kidney tissue and promote the formation of calcium-containing crystals (31). The long-term accumulation of these compounds in the kidneys could exacerbate the risk of stone formation, especially in individuals exposed to high levels of these substances over prolonged periods.

The interaction between PFAS exposure and sleep duration likely amplifies these biological processes. Sleep disturbances have been shown to influence the balance of minerals like calcium, phosphate, and oxalate, which are critical in kidney stone formation (32, 33). The synergistic effect of PFAS exposure and inadequate sleep may disrupt this delicate mineral balance more profoundly, leading to an increased likelihood of kidney stone development. In particular, insufficient sleep could exacerbate the nephrotoxic effects of PFAS, further heightening the risk of kidney damage and stone formation (34). This interaction emphasizes the need for integrated public health strategies that address both environmental exposures and lifestyle factors in reducing kidney stone prevalence.

This study also has notable strengths, including the large and diverse NHANES cohort and the rigorous statistical models used to account for potential confounding factors. However, there are limitations that must be considered. The cross-sectional design of the study precludes the establishment of causality, and further longitudinal studies are needed to confirm the temporal relationship between PFAS exposure, sleep duration, and kidney stone formation (35). Additionally, the reliance on self-reported sleep duration may introduce measurement bias, as sleep patterns may not be accurately captured through subjective reporting. Furthermore, sleep quality, which is a known predictor of metabolic health and kidney function, was not assessed in this study. The lack of objective measures, such as actigraphy, further limits the accuracy of sleep duration reporting. These methodological gaps could lead to residual confounding and measurement error, which should be considered when interpreting the findings. Moreover, PFAS exposure was measured using blood concentrations of various perfluoroalkyl compounds, which reflect short-term exposure (weeks to months). It is important to acknowledge that this measure may underestimate the long-term cumulative effects of PFAS exposure, which could be more relevant for nephrolithiasis pathogenesis. Long-term exposure, potentially assessed through more comprehensive biomarkers or repeated measurements over time, would provide a more accurate reflection of cumulative PFAS effects on kidney health. Previous research has indicated a potential association between lipid-related covariates and the development of kidney stones (36–38). However, these lipid-related covariates were not incorporated into the present study, necessitating further exploration and discussion in subsequent investigations. Finally, while we controlled for several important confounders, residual confounding by unmeasured variables, such as dietary habits or specific medications, cannot be ruled out (39).

Future research should focus on elucidating the underlying mechanisms that explain the synergistic effects of PFAS exposure and sleep duration on kidney stone formation. Longitudinal studies are essential to establish causal relationships and to investigate whether interventions aimed at reducing PFAS exposure or improving sleep quality can reduce the incidence of kidney stones. Moreover, experimental studies should explore the biochemical pathways through which PFAS disrupt mineral metabolism and how these disruptions contribute to the formation of kidney stones. Ultimately, a better understanding of these complex interactions will help inform more effective public health strategies and regulatory policies aimed at reducing kidney stone risk.

## 5 Conclusion

In conclusion, this study highlights the importance of both environmental exposures and lifestyle factors in the development of kidney stones. Our findings underscore the need for a comprehensive approach to kidney stone prevention that includes both reducing PFAS exposure and promoting healthy sleep habits. By addressing these two critical factors, it may be possible to reduce the burden of kidney stones, a condition that significantly impacts public health worldwide.

## Data Availability

The datasets presented in this study can be found in online repositories. The names of the repository/repositories and accession number(s) can be found in the article/supplementary material.
